# Improving Management of Viral Febrile Illness and Reducing the Need for Empiric Antibiotics Using VIDAS^®^ Immunoassay for Dengue and Chikungunya: A West African Multicentric Study

**DOI:** 10.3390/diagnostics15172269

**Published:** 2025-09-08

**Authors:** Fanette Ravel, Solenne Robert, Diakourga Arthur Djibougou, Kigninlman Horo, Aristophane Tanon, Privat Ango, Palpouguini Félix Lompo, Faustine Meynier, Ludovic Brossault, Umit Guler, Jacques Simpore, Potiandi Serge Diagbouga

**Affiliations:** 1 bioMérieux SA, 69280 Marcy l’Etoile, France; fanette.ravel@biomerieux.com (F.R.); ludovic.brossault@biomerieux.com (L.B.); umit.guler@biomerieux.com (U.G.); 2 Institut de Recherche en Sciences de la Santé (IRSS), Centre National de la Recherche Scientifique et Technologique (CNRST), Ouagadougou 01 BP 641, Burkina Faso; lynndjibougou@gmail.com (D.A.D.); felixlompo@gmail.com (P.F.L.); 3 Unité de Formation et de Recherche en Sciences Médicales, Université Félix Houphouët-Boigny d’Abidjan, Abidjan 00225, Côte d’Ivoire; kigninlmanh@yahoo.fr (K.H.); angoprivat@gmail.com (P.A.); 4 Service des Urgences du CHU de Cocody, UFR Sciences Médicales—Université Félix Houphouët Boigny, Abidjan 00225, Côte d’Ivoire; aristotanon@gmail.com; 5 bioMérieux SA, 38000 Grenoble, France; faustine.meynier@biomerieux.com; 6 Centre de Recherche Biomoléculaire Piétro Anigoni (CERBA), Ouagadougou 01 BP 364, Burkina Faso; simpore93@gmail.com

**Keywords:** dengue, chikungunya, diagnosis, VIDAS^®^, differential diagnosis, antibiotic use, West Africa

## Abstract

**Background:** Dengue and chikungunya are endemic in West Africa, posing significant public health issues. The aim of this study was to evaluate the impact of differential and systematic diagnosis of dengue and chikungunya on patient management and on antibiotic use in Burkina Faso and Ivory Coast. **Methods**: A multicenter prospective cohort study was conducted in both countries involving patients with suspected dengue and/or chikungunya viremia. VIDAS^®^ diagnostic tests (bioMérieux SA, Marcy-l’Étoile, France) were provided to the intervention sites, while the control sites initially followed standard of care before testing at the end of the study. The primary outcome was defined as antibiotic prescription or non-initiation/discontinuation, and the secondary endpoints included hospital resource use, patient satisfaction, and health-related quality of life (HRQoL), analyzed through Chi-square and logistic regression using SAS software v9.4. **Results**: Out of 775 enrolled patients, 767 had corresponding VIDAS^®^ Dengue and VIDAS^®^ Chikungunya results, with 570 having recorded antibiotic therapy (initiated, non-initiated or discontinued). Both Burkina Faso and Ivory Coast observed an increase in antibiotic discontinuation (or non-initiation) rates at the intervention sites compared to control sites: increased from 60% to 78% in Burkina Faso and from 36% to 83% in Ivory Coast. Hospitalization rates within seven days following inclusion were also lower in intervention sites than in the control sites: Burkina Faso 41% as compared with 97% and Ivory Coast 24% as compared with 98%. Patient-reported antibiotic use within seven days post-inclusion was also significantly lower in intervention sites. **Conclusions**: The results showed a reduction in potential antibiotic overuse and hospital admissions (i.e., hospitalization rates within seven days) in both the Burkina Faso and Ivory Coast interventions sites. These findings emphasize the importance of enhanced diagnostic strategies for the improvement of patient outcomes and the fight against antibiotic resistance. This study also highlights the need for implementing systematic and differential diagnosis of dengue and chikungunya in West Africa where febrile infections are endemic. Further studies are warranted to explore the economic benefits of these diagnostic strategies.

## 1. Introduction

Febrile illnesses are highly prevalent and represent one of the leading causes of medical consultations in West Africa. These conditions are characterized by the onset of a fever, which may be triggered by several infectious diseases including leptospirosis, malaria and arboviruses, most notably dengue and chikungunya [1].

Dengue is the most common arboviral infection in humans and the most widespread mosquito-transmitted disease worldwide [2,3]. It is predominantly found in Asia’s tropical belt, Latin America, the Western Pacific and Africa, all of which are endemic or hyperendemic regions [2]. The global incidence of dengue is rising and is now classified as a ‘re-emerging’ disease [4]. According to the World Health Organisation (WHO), an estimated 390 million dengue infections occur annually (95% confidence interval: 284–528 million), of which 96 million (67–136 million) presents with clinical symptoms of varying severity [5,6].

Dengue is caused by an arbovirus belonging to the *Flaviviridae* family comprising four distinct serotypes: DENV-1, DENV-2, DENV-3 and DENV-4. Immunity acquired in response to infection with one of the serotypes confers protective immunity only against the infecting serotype but not against other serotypes [4,5,6]. In endemic regions, individuals are frequently infected with multiple serotypes over time, which increases the risk of developing severe dengue. The pathophysiological mechanisms underlying severe dengue remain poorly understood but are believed to involve complex interactions between immunological, genetic, and viral factors [7]. According to the 2009 WHO classification, the course of dengue fever is divided into two categories: DENV infection with and without warning signs [6].

Chikungunya, on the other hand, is a debilitating disease caused by the chikungunya virus and transmitted to humans by *Aedes* mosquitoes [8]. Chikungunya spread to over 100 countries across Africa, Asia, Europe, and the Americas, with numerous outbreaks affecting millions of individuals [8,9,10,11,12]. The growing geographic spread of chikungunya is likely multifactorial, driven by viral and vector adaptation to environmental changes, urbanization, changes in human behavior, and increased global travel [8,13].

Dengue has emerged as an important arboviral disease in West Africa, with increasing reported cases and evidence of endemic transmission in several countries, including Burkina Faso and Ivory Coast [14,15]. Chikungunya outbreaks have been reported periodically, with increasing geographic spread and outbreaks documented in 2023 [15]. Malaria also remains a leading cause of morbidity and mortality in both Burkina Faso and Ivory Coast, with high endemicity in rural and urban settings [16].

Dengue and chikungunya often co-circulate in the same endemic regions, and co-infections have been documented [13,17,18,19,20]. Early clinical symptoms of both infections are often indistinguishable and non-specific, typically including fever, headache, myalgia, arthralgia, and maculopapular rash [13,17,21]. Climate change is also altering the epidemiology of various arboviruses, making it imperative for healthcare professionals in all regions to be familiar with these diseases [22]. Moreover, because symptoms overlap with other febrile illnesses such as malaria, leptospirosis, typhoid, and given the limited access to diagnostic tools and inadequate surveillance systems, the true burden of dengue and its socioeconomic impact are likely underestimated in the region of West Africa [23].

In the absence of accurate diagnosis, healthcare providers often resort to empirical antibiotic prescriptions, even when the underlying infection is viral, contributing to unnecessary antibiotic use. This practice is further compounded by self-medication with over-the-counter antibiotics. Such misuse contributes to the growing threat of antimicrobial resistance in sub-Saharan Africa [24], undermines antibiotic effectiveness, and potentially compromises patient outcomes in the case of bacterial infections. These inappropriate practices increase the risk of avoidable morbidity and mortality associated with febrile illnesses in endemic settings.

The aim of this multicenter study was to assess the impact of differential and systematic diagnosis of dengue and chikungunya on patient management and, particularly, on antibiotic use in Burkina Faso and Ivory Coast. This study highlights the need for implementing systematic and differential diagnosis of dengue and chikungunya in West Africa where febrile infections are endemic.

## 2. Material and Methods

### 2.1. Study Design

The primary objective of this multicenter study was to evaluate the impact of differential and systematic diagnosis of dengue and chikungunya using the VIDAS^®^ Dengue NS1 Ag, VIDAS^®^ Anti-Dengue IgM, VIDAS^®^ Anti-Dengue IgG, VIDAS^®^ Anti-Chikungunya IgM and VIDAS^®^ Anti-Chikungunya IgG (bioMérieux SA, Marcy-l’Étoile, France) on adult patient management and antibiotic prescription rates (discontinuation or non-initiation) presenting symptoms compatible with dengue or chikungunya.

The secondary objectives were to evaluate the impact of implementing differential diagnosis of dengue and chikungunya testing on hospital resource utilization (i.e., number of hospital days and visits within seven days following clinical management by the healthcare establishment), as well as on patient reported outcomes such as quality of life and satisfaction. Patient satisfaction was assessed at the point of clinical consultation for outpatients and at discharge for inpatients using a 5-point scale. Quality of life was measured at inclusion and again seven days post-inclusion, and productivity loss was evaluated based on the number of workdays lost in the seven days following clinical care.

In each of the two countries included in the study, two CHUs were selected based on their comparable size, historical data on reported dengue cases in previous years, and their similar practices in the management of dengue in accordance with the Ministry of Health’s standards. The assignment of intervention versus control sites was carried out randomly.

At the intervention sites, patients were tested for dengue and chikungunya using VIDAS^®^ assays. In addition, an awareness campaign was implemented to promote correct use of the VIDAS^®^ diagnostic tools and to highlight the importance of differential diagnosis in patients presenting with febrile illness compatible with dengue or chikungunya symptoms, prior to definitive laboratory diagnosis. Final diagnosis at these sites was based on both clinical presentation and biological results from the VIDAS^®^ dengue and chikungunya assays.

At the control sites, patients received the national standard of care, which included clinical diagnosis based on symptoms and, when appropriate, the use of routinely available diagnostic tests, mainly rapid diagnostic tests (RDTs). At the end of the recruitment period, all samples control sites were tested using VIDAS^®^ assays to retrospectively estimate the number of dengue and chikungunya cases that had been undiagnosed or misdiagnosed under standard care conditions.

### 2.2. Study Cohort and Blood Samples

**Figure 1:**
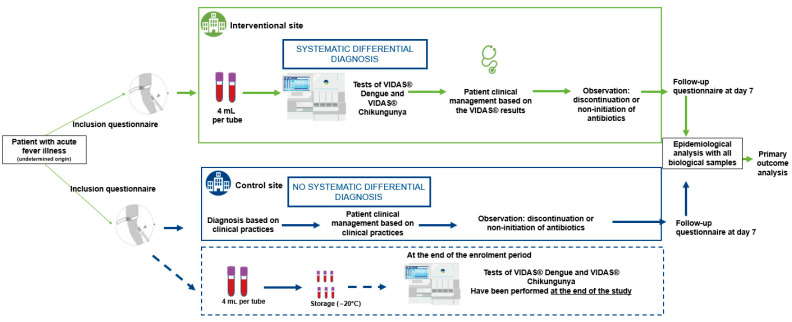
Study workflow diagram.

Samples and socio-clinical data were collected between August 2023 and October 2023 from adult patients with suspected dengue and/or chikungunya infections. These patients presented at the hospital emergency department with fever (≥38.5 °C) and one or more of the following symptoms: headache, retro-orbital pain, myalgia, arthralgia, abdominal pain, nausea, vomiting, adenopathy, skin rash, spontaneous bleeding (purpura, epistaxis, hematemesis, etc.) and asthenia (Figure 1). Samples were collected prospectively in Ivory Coast and Burkina Faso and tested in laboratories at two distinct sites (Table 1).

Collected blood samples (two samples of 4 mL per patient) were aliquoted and then tested with the different assays. For site 3, fresh aliquots were tested directly at the site. For site 1, aliquots were frozen at the collection site, transported frozen under controlled conditions to the testing site. For site 2 and site 4, aliquots were frozen at the collection site, transported frozen under controlled conditions, and stored frozen at −20 °C until testing at the end of the study.

### 2.3. Ethical Considerations

Informed consent was obtained from all subjects involved in the study. Study protocols were approved by the institutional review board at each institution (Comité d’éthique institutionnel de l’IRSS (N° A043-2022/CEIRES/IRSS) and Comité d’Ethique pour la Recherche en Santé (CERS) of Ministry of Health for Burkina Faso (N° 2022-11-230) and Comité National d’Ethique des Sciences de la Vie et la Santé of Ministry of Health for Ivory Coast (N° 163-22/MSHPCMU/CNESVS-km)). The study was also registered as a clinical trial under the registration number (CTRN): NCT06257810.

### 2.4. VIDAS^®^ Assays

The VIDAS^®^ Dengue NS1 Ag, VIDAS^®^ Anti-Dengue IgM, and VIDAS^®^ Anti-Dengue IgG assays (bioMérieux SA, Marcy l’Étoile, France) are automated qualitative two-step immunoassays developed for use on VIDAS^®^ instruments [25]. The VIDAS^®^ Dengue NS1 Ag assay detects the dengue NS1 antigen of the four DENV serotypes (DENV-1, DENV-2, DENV-3 and DENV-4). They are intended as an aid in the diagnosis of patients with clinical symptoms consistent with dengue infection. The VIDAS^®^ Anti-Dengue IgM and IgG assays detect IgM and IgG antibodies, respectively, recognizing antigens of the four DENV serotypes, owing to the use of a recombinant tetravalent EDIIIT2 protein composed of the antigenic DENV-specific envelope domain III of the four DENV serotypes [25,26].

VIDAS^®^ Anti-Chikungunya IgM and VIDAS^®^ Anti-Chikungunya IgG (bioMérieux SA, Marcy-l’Étoile, France) are automated qualitative two-step immunocapture assays combined with enzyme-linked fluorescent assay (ELFA) detection, developed for the VIDAS^®^ family of instruments. The development of these assays was made possible using technology and methods developed at the United States National Institute of Allergy and Infectious Diseases, Vaccine Research Center. They are intended as an aid in the diagnosis of patients with clinical symptoms consistent with chikungunya infection. The Solid Phase Receptacle (SPR) serves as the solid phase as well as the pipetting device. Reagents for the assay are ready-to-use and pre-dispensed in the sealed reagent strip. All steps are performed automatically by the instrument and completed within approximately 40 min.

The three VIDAS^®^ dengue diagnostic tests and the two VIDAS^®^ chikungunya diagnostic tests were performed and interpreted according to the instructions for use, as previously described [25].

For both dengue and chikungunya, a laboratory-confirmed case was defined as a patient presenting with compatible clinical symptoms and a positive result in at least one of the VIDAS^®^ assays, except for isolated IgG positivity. For dengue: NS1 antigen and/or Anti-Dengue IgM and/or Anti-Dengue IgG. For chikungunya: Anti-Chikungunya IgM and/or Anti-Chikungunya IgG. Isolated IgG positivity, in the absence of IgM or NS1 antigen for dengue and in the absence of IgM for chikungunya, was interpreted as past or secondary infection and considered in the clinical context.

### 2.5. Patients’ Clinical Follow-Up

Patients were contacted by telephone seven days after inclusion to assess the progression of their illness, inquire about the use of any non-prescribed antibiotics, determine whether they had been hospitalized, and collect information on the number of days they had missed from work since discharge.

### 2.6. Statistical Analysis

The primary endpoint was a composite endpoint, defined by either the discontinuation or non-initiation of antibiotics. The proportion of patients with either antibiotic discontinuation or non-initiation was monitored and compared between sites (intervention versus (vs.) control) within each country, using a Chi-square test. A single statistical test was applied on this composite endpoint, thereby avoiding issues of multiplicity, and no statistical adjustments were necessary.

Baseline patient characteristics, including age, gender, professional treatment (Yes/No), individual symptoms (Yes/No), time from symptom onset to baseline, and the impact of symptoms on daily activities and/or work (Yes/No), were compared between sites using Chi-square tests (or Fisher’s exact test when cell counts were insufficient) for categorical variables and Student’s *t*-test (or Wilcoxon-Mann–Whitney tests when normality distribution could not be assumed) for continuous variables. A multivariate logistic regression model was then used to adjust for observed baseline significant differences between the two cohorts.

Secondary endpoints, including antibiotic use reported by the patient, hospitalization, the proportion of patients who missed workdays, the number of missed workdays (among patients with professional treatment), and patient satisfaction, were analyzed using the same statistical methods as for the primary endpoint.

Results are reported as univariate and adjusted odds ratio (OR) for endpoints with 2 categorical values or incidence rate ratio (IRR) and for endpoints with more than 2 categorical or continuous values with 95% Confidence Intervals (95% CI). When value 1.0 is outside the 95% CI of the OR or the IRR, the association between the response and the site is statistically significant (alpha risk at 5% level), meaning that there is a significant difference in the endpoint results between both sites. On the other hand, when value 1.0 is within the 95% CI of the OR or IRR, the association between the response and the site is not shown (alpha risk at 5% level), meaning that the difference in the endpoint results between both sites is not significant.

All analyses assumed that the prevalence of dengue and chikungunya was similar between the two study sites (intervention vs. control). To verify this assumption, a preliminary comparison of prevalence rates between the sites within each country was performed using a Chi-square test.

In all analyses conducted, *p*-values ≤ 5% were considered statistically significant. All analyses were conducted and interpreted using SAS software v9.4.

## 3. Results

**Figure 2:**
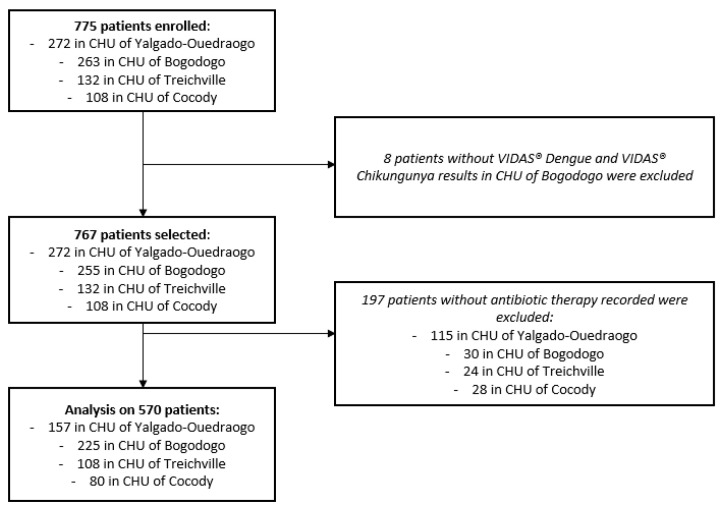
Patient inclusion flowchart.

Among the 775 patients enrolled from hospital emergency departments, 767 had corresponding VIDAS^®^ Dengue and VIDAS^®^ Chikungunya results, 570 of which had a recorded antibiotic therapy discontinuation or non-initiation. Thus, the final analysis was performed on 570 patients: 382 in Burkina Faso (157 (41%) in the control site and 225 (59%) in the intervention site) and 188 in Ivory Coast (108 (57%) in the control site and 80 (43%) in the intervention site) (Figure 2).

### 3.1. Global Results

Global results from all sites are presented below (Table 2).

### 3.2. Baseline Clinical and Demographic Characteristics

Dengue or chikungunya infection was determined based on positive VIDAS^®^ results for all sites. However, only 19 chikungunya infections were detected in total (10 in Burkina Faso and 9 in Ivory Coast). Therefore, the combined prevalence of these two arboviruses was calculated and compared between the sites for each country. The combined prevalence was 48% in Burkina Faso (50% in control site (79 out of 157 patients with laboratory-confirmed dengue and/or chikungunya) and 47% in intervention site (104 out of 225 patients with laboratory-confirmed dengue and/or chikungunya)) and 43% in Ivory Coast (43% in control site (46 out of 108 patients with laboratory-confirmed dengue and/or chikungunya) and 43% in intervention site (34 out of 80 patients with laboratory-confirmed dengue and/or chikungunya)). No difference was shown between the sites within each country (*p*-value = 0.5617 for Burkina Faso and 0.9899 for Ivory Coast).

For both countries, no difference in dengue and/or chikungunya prevalence were shown between the two sites (*p*-value is 0.5617 for Burkina Faso and 0.9899 for Ivory Coast), indicating no adjustment was required for the analysis that was performed. However, significant differences were noted in patient characteristics such as socio-demographic and/or clinical factors at baseline) between the two sites in each country. To account for potential bias due to these baseline differences, a multivariate analysis was conducted to adjust the model for the significant factors.

In Burkina Faso, the study population had a median age of 33 years (range: 18–99 years), with 195 males (51%) and 187 females (49%). Of those participants, 284 (74%) had a professional treatment and 136 (36%) had used antibiotics (prescribed or self-medicated) in the 10 days prior to inclusion.

In Ivory Coast, the median age was also 33 years (range: 18–94 years), with 93 males (49%) and 95 females (51%). Among them, 77 (41%) had a professional treatment and 77 (41%) had used antibiotics (prescribed or self-medicated) in the 10 days prior to inclusion.

In Burkina Faso, the duration between symptoms’ onset and admission to emergency department was between 0 and 2 days for 71 (19%) participants, between 3 and 6 days for 199 (52%) participants and between 7 and 10 days for 112 (29%) participants. In Ivory Coast, the duration was between 0 and 2 days for 23 (12%) participants, between 3 and 6 days for 107 (57%) participants and between 7 and 10 days for 57 (30%) participants. The symptoms observed at inclusion are presented below (Table 3).

### 3.3. Rate of Antibiotic Prescription (Discontinuation or Non-Initiation)

**Figure 3:**
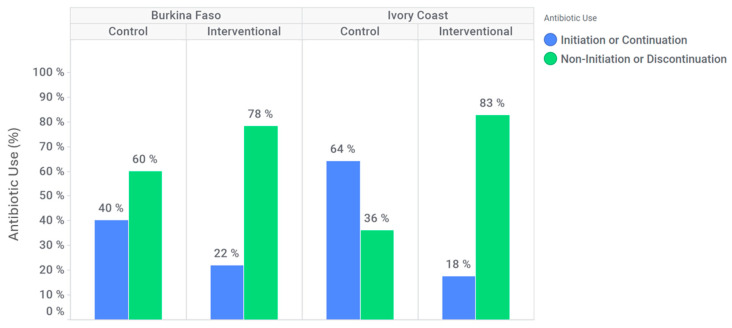
Rate of antibiotic discontinuation or non-initiation per country.

In Burkina Faso, antibiotic discontinuation or non-initiation occurred in 94 (60%) participants at the control site, compared to 176 (78%) participants at the intervention site (Figure 3). At the intervention site, the likelihood of antibiotic discontinuation or non-initiation was higher than in the control site (adjusted OR: 1.6 [95% CI: 0.9–2.8]). Additionally, among patients who tested positive for dengue and/or chikungunya, antibiotic discontinuation or non-initiation occurred in 51 (65%) participants at the control site against 84 (80%) participants at the intervention site. Indeed, in this subgroup, for Burkina Faso, discontinuation or non-initiation of antibiotics is significantly higher at the intervention site compared to the control site (adjusted OR: 2.0 [95% CI: 0.8; 4.7]).

In Ivory Coast, an antibiotic discontinuation or non-initiation was observed for 39 (36%) participants at the control site against 66 (83%) participants in intervention site (Figure 3). At the intervention site, discontinuation or non-initiation of antibiotics is significantly higher (adjusted OR: 5.4 [95% CI: 1.9; 15.2]). Furthermore, among patients who tested positive for dengue and/or chikungunya only, antibiotic discontinuation or non-initiation was effective for 16 (35%) participants at the control site against 30 (88%) participants at the intervention site. Indeed, in this subgroup, discontinuation or non-initiation of antibiotics is significantly higher at the intervention site compared to the control site (adjusted OR: 20.3 [95% CI: 1.9; 220.8]).

### 3.4. Antibiotic Use Reported by the Patient

In Burkina Faso, 41 (27%) participants at the control site reported antibiotic use, including use of prescribed antibiotics and self-medication, within seven days following inclusion, compared to 19 (8%) participants at the intervention site. In Ivory Coast, 65 (61%) participants at the control site reported antibiotic use, including use of prescribed antibiotics and self-medication, within seven days following inclusion compared to 15 (20°%) participants at the intervention site. For both countries, the use of antibiotics by the patient within seven days following inclusion was significantly lower at the intervention site compared to the control site (adjusted OR: 0.2 [95% CI: 0.1; 0.5] for Burkina Faso and 0.2 [95% CI: 0.1; 0.6] for Ivory Coast).

### 3.5. Hospitalization

**Figure 4:**
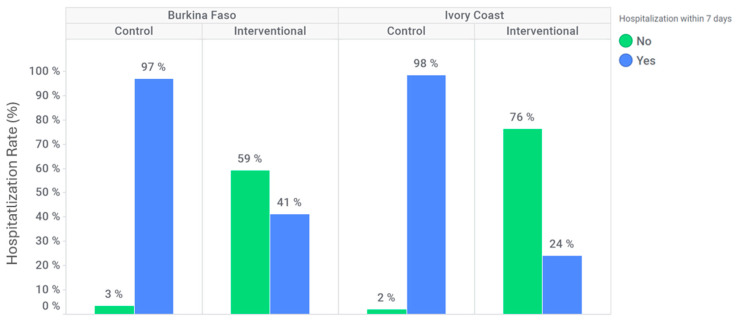
Hospitalization within 7 days after inclusion.

In Burkina Faso, 152 (97%) participants were hospitalized within seven days after inclusion at the control site compared to 92 (41%) participants at the intervention site (Figure 4). In Ivory Coast, 106 (98%) participants were hospitalized within seven days after inclusion at the control site compared to 19 (24%) participants at the intervention site (Figure 4). The hospitalization rate within seven days following inclusion was significantly lower at the intervention site for both countries (adjusted OR: 0.026 [95% CI: 0.009; 0.074] for Burkina Faso and 0.003 [95% CI: 0.000; 0.036] for Ivory Coast).

### 3.6. Number of Missed Workdays (Among Patients with a Professional Activity)

In Burkina Faso, 51 (56%) participants missed workdays within seven days after inclusion at the control site against 42 (22%) at the intervention site. In Ivory Coast, 16 (59%) participants missed workdays within seven days after inclusion at the control site against 24 (51%) at the intervention site. The proportion of patients that missed workdays within seven days after inclusion was significantly lower at the intervention site for Burkina Faso compared to the control site (adjusted OR: 0.2 [95% CI: 0.1; 0.3]), but no difference was shown for Ivory Coast (adjusted OR: 1.8 [95% CI: 0.5; 6.7]). Moreover, the Incidence Rate Ratio (IRR) showed that number of missed workdays was significantly shorter in the intervention site for Burkina Faso (IRR: 1.8 [95% CI: 1.4; 2.3]). On the other hand, no difference was observed between the two sites in Ivory Coast regarding the number of missed workdays (IRR: 1.0 [95% CI: 0.6; 1.5]).

### 3.7. Patient Satisfaction Evolution Between Inclusion and Day 7

In both countries, no difference was observed between the two sites in the evolution of patient satisfaction regarding clinical management by healthcare professionals from inclusion to seven days after inclusion (*p*-values > 5% for both countries).

### 3.8. Quality of Life Evolution Between Inclusion and Day 7

In both countries, no difference was observed between the two sites in the evolution of quality of life from inclusion to seven days after inclusion (*p*-values > 5% for both countries).

### 3.9. Coinfection

In Ivory Coast, 173 patients were tested for malaria using the HWTAi Dengue IgG/IgM/NS1 Combo Rapid Test. Among these, 96 patients tested positive for malaria, and 43 (45%) were co-infected with dengue and/or chikungunya. Data on co-infection was not collected for patients at the Burkina Faso sites.

## 4. Discussion

This multicenter study evaluated the impact of differential and systematic diagnosis of dengue and chikungunya on patient management and antibiotic prescription in Burkina Faso and Ivory Coast. Both countries showed an increase in antibiotic discontinuation or non-initiation rates at the intervention sites compared to the control sites. In addition, the relatively high rate of treatment initiation or continuation observed in the control groups of both countries may be explained by the fact that the standard of care does not include specific diagnostic tools for dengue or chikungunya. Consequently, clinicians often relied on hematological results and clinical presentation, which may mimic bacterial infections, leading to empirical antibiotic prescriptions.

Thus, the study provides valuable insights into the systematic differential diagnosis of arboviruses in West Africa, an area with limited publications and scarce epidemiological and prevalence data on these infections [27].

Most arboviruses are endemic in resource-limited countries, where their burden contributes significantly high levels of morbidity and mortality [28]. However, this burden remains poorly understood due to several factors. The overlap in clinical symptoms between arbovirus infections and other tropical febrile illnesses, such as malaria, complicated accurate diagnosis and differentiation. Moreover, the limited capacity of laboratories and the insufficient knowledge of healthcare workers regarding arboviruses hinder the prompt detection and confirmation of infections, delaying case reporting and the implementation of preventive measures [27].

As of today, serological antibody tests are available in most African countries and are used during outbreaks as well as in laboratories with limited technical facilities. These tests are employed during outbreaks or in suspected cases that could indicate an outbreak, but they are still not routinely included test in diagnostic protocols in the majority of African countries [29]. The benefits of accurate diagnosis include improved detection of infections within the population, faster access to appropriate clinical management and treatment, and the prevention of inappropriate use of medications such as antimalarials and antibiotics. In a context of limited resources, these measures could not only enhance the management of febrile illnesses but also help reduce the overall burden of disease [30].

Moreover, West Africa has the highest reported prevalence of self-medication with antibiotics, reaching up to 70.1% [31]. The results of this study indicate that the use of antibiotics by patients within seven days following inclusion was significantly lower at the intervention site compared to the control sites. This underscores the importance of differential and systematic diagnosis in reducing potential antibiotic overuse or uncontrolled use, which can, in turn, help limit antibiotic resistance. Additionally, early differential diagnosis of arboviruses can positively impact hospitalization duration. Specifically, we observed that the hospitalization rate within seven days following inclusion was significantly lower at the intervention site.

The proportion of patients who missed workdays within seven days after inclusion was significantly lower at the intervention site in Burkina Faso compared to the control site. This finding suggests that systematic differential diagnosis may have an economic impact related to patients’ work capacity, an aspect that could be further explored in a medico-economic study.

Additionally, the highly mobile lifestyle of the population, the increased accessibility provided by global transportation networks, and climate change are expected to contribute to a rise in co-infection, particularly with dengue and malaria [32]. In fact, the 45% prevalence of co-infection (dengue/chikungunya and malaria) in Ivory Coast highlights the importance of systematic diagnosis of arboviruses, alongside malaria testing, in endemic areas of West Africa. This is crucial for providing the most appropriate clinical management, especially in regions where co-circulation of these diseases is highly prevalent.

There are several limitations to this study. Despite being standard practice, malaria testing was not conducted in Burkina Faso for patients presenting with fever of unknown origin. The reason for the missing data (e.g., results not provided or tests unavailable) remain unclear, and as a result, malaria data were not included in the overall analyses. Only limited malaria prevalence data are available for Ivory Coast. For these reasons, it is not possible to extrapolate regarding the expected number of co-infections in either country. Additionally, the study was limited to two West African countries, with more participants enrolled in Burkina Faso due to epidemic periods (e.g., dengue and chikungunya outbreaks), leading to a reduced sample size in Ivory Coast. Some subgroups analyses were based on a few as thirty patients, which is too small a sample to draw definitive conclusion. Furthermore, the awareness campaign was implemented exclusively at the intervention sites, which may have introduced bias in the results. Expanding the analysis to include more countries, or exploring differences between public and private health centers, or including the pediatric population, would provide valuable insights. Longitudinal studies that monitor long-term outcomes and antibiotic resistance patterns following the implementation of structured diagnostic protocols would also be beneficial. Also, the higher adjusted OR in Ivory Coast may reflect differences in healthcare practices, clinician awareness, or baseline antibiotic prescribing behaviors. Further investigation into local clinical protocols and diagnostic capacities is warranted.

Despite these limitations, the study significantly contributes to scientific knowledge on tropical fevers like dengue and chikungunya, as well as their diagnosis by health systems, particularly in Ivory Coast and Burkina Faso, and more broadly in West Africa.

## 5. Conclusions

This study aimed to assess the impact of differential and systematic diagnosis of dengue and chikungunya on patient management and antibiotic use in Burkina Faso and Ivory Coast. The findings underscore the value of improved diagnostic strategies in optimizing patient management and reducing unnecessary antibiotic prescriptions, thereby supporting better clinical and public health outcomes, and the critical role of systematic and differential diagnostics in managing viral infections like dengue and chikungunya, especially in resource-limited settings. By reducing unnecessary antibiotic use and hospitalizations, the study highlights the potential for improved patient care, decreased potential antibiotic overuse and, ultimately, a reduction in antibiotic resistance. Alongside these findings, there is a clear need for further evidence on the economic benefits of differential diagnosis tools in West African health centers. Demonstrating the medico-economic advantages of such tools could encourage government support for health programs.

## Figures and Tables

**Table 1:** Description of study sites.

Site	Collection Site	Type	Samples	Testing Site
1	CHU of Bogodogo, Ouagadougou, Burkina Faso	Intervention	Prospective cohort	CERBA, Ouagadougou, Burkina Faso
2	CHU of Yalgado-Ouedraogo, Ouagadougou, Burkina Faso	Control	Prospective cohort	CERBA, Ouagadougou, Burkina Faso
3	CHU of Cocody, Abidjan, Ivory Coast	Intervention	Prospective cohort	CHU of Cocody, Abidjan, Ivory Coast
4	CHU of Treichville, Abidjan, Ivory Coast	Control	Prospective cohort	CHU of Cocody, Abidjan, Ivory Coast

**Table 2:** Global results.

	Burkina Faso			Ivory Coast		
	*N*	Univariate Analysis	Multivariate Analysis	*N*	Univariate Analysis	Multivariate Analysis
		OR/IRR [95% CI]			OR/IRR [95% CI]	
Antibiotic non-initiation or discontinuation rate as reported by physician prescriptions	382	2.4 [1.5; 3.8]	1.6 [0.9; 2.8]	188	8.3 [4.2; 16.8]	5.4 [1.9; 15.2]
Antibiotic non-initiation or discontinuation rate in patients that test positive for dengue and/or chikungunya using VIDAS^®^ diagnostic tests	183	2.1 [1.1; 4.1]	2.0 [0.8; 4.7]	80	14.1 [4.2; 47.0]	20.3 [1.9; 220.8]
Antibiotic use as reported by the patient within seven days following inclusion, including use of prescribed antibiotics and self-medication	378	0.3 [0.1; 0.5]	0.2 [0.1; 0.5]	182	0.2 [0.1; 0.3]	0.2 [0.1; 0.6]
Hospitalization rate within seven days following inclusion	382	0.023 [0.009; 0.058]	0.026 [0.009; 0.074]	188	0.006 [0.001; 0.026]	0.003 [0.000; 0.036]
Proportion of patients that have missed workdays within seven days after inclusion (amongst patient with a professional activity)	282	0.2 [0.1; 0.4]	0.2 [0.1; 0.3]	74	0.7 [0.3; 1.9]	1.8 [0.5; 6.7]
Number of missed workdays (amongst patients with a professional activity)`	281	0.7 [0.5; 0.8]	1.8 [1.4; 2.3]	74	0.7 [0.5; 0.9]	1.0 [0.6; 1.5]
Evolution of patient satisfaction with clinical management between day 1 and day 7—Staff	377	0.6 [0.4; 0.9]	0.6 [0.3; 1.1]	182	1.3 [0.7; 2.5]	1.4 [0.5; 3.9]
Evolution of patient satisfaction with clinical management between day 1 and day 7—Diagnostic	375	0.5 [0.3; 0.8]	0.6 [ 0.3; 1.1]	153	1.9 [0.9; 4.0]	2.0 [0.6; 6.4]
	***N***	**Average difference between day 7 and day 1** **[95% CI]**		***N***	**Average difference between day 7 and day 1** **[95% CI]**	
Quality of life evolution between day 1 and day 7	378	0.00 [−0.02; 0.03]	0.02 [−0.01; 0.06]	182	0.11 [0.07; 0.15]	0.05 [−0.01; 0.10]

OR: Odds Ratio/IRR: Incidence Rate Ratio/95% CI: 95% Confidence Intervals.

**Table 3:** Symptoms observed at inclusion.

Symptoms	Burkina Faso	Ivory Coast
Cephalalgia	345 (90%) participants	178 (95%) participants
Retro-orbital pain	166 (43%) participants	123 (65%) participants
Myalgia and arthralgia	278 (73%) participants	175 (93%) participants
Nausea	161 (42%) participants	123 (65%) participants
Vomiting	163 (43%) participants	104 (55%) participants
Lymphadenopathy	22 (6%) participants	36 (19%) participants
Rash	22 (6%) participants	16 (9%) participants
Abdominal pain	174 (46%) participants	84 (45%) participants
Asthenia	359 (94%) participants	181 (96%) participants
Spontaneous bleeding	55 (14%) participants	12 (6%) participants

## Data Availability

The data presented in this study are available within the article.

## Abbreviations

The following abbreviations are used in this manuscript:

HRQoL	Health-related quality of life
WHO	World Health Organization
RDT	Rapid Diagnostic Test
ELFA	Enzyme-linked fluorescent assay
SPR	Solid phase receptacle
OR	Odds ratio
IIR	Incidence rate ratio
CI	Confidence intervals
CHU	Centre Hospitalier Universitaire
